# The influenza replication blocking inhibitor LASAG does not sensitize human epithelial cells for bacterial infections

**DOI:** 10.1371/journal.pone.0233052

**Published:** 2020-05-15

**Authors:** Janine J. Wilden, Andre van Krüchten, Lutz Gieselmann, Eike R. Hrincius, Stefanie Deinhardt-Emmer, Karoline F. Haupt, Hannah F. Preugschas, Silke Niemann, Stephan Ludwig, Christina Ehrhardt

**Affiliations:** 1 Institute of Virology Muenster (IVM), Westfaelische Wilhelms-University (WWU) Muenster, Germany; 2 Section of Experimental Virology, Institute of Medical Microbiology, Jena University Hospital, Jena, Germany; 3 Institute of Medical Microbiology, Westfaelische Wilhelms-University (WWU) Muenster, Germany; 4 Cluster of Excellence EXC 1003 “Cells in Motion”, WWU Muenster, Germany; University of South Dakota, UNITED STATES

## Abstract

Severe influenza virus (IV) infections still represent a major challenge to public health. To combat IV infections, vaccines and antiviral compounds are available. However, vaccine efficacies vary with very limited to no protection against newly emerging zoonotic IV introductions. In addition, the development of resistant virus variants against currently available antivirals can be rapidly detected, in consequence demanding the design of novel antiviral strategies. Virus supportive cellular signaling cascades, such as the NF-κB pathway, have been identified to be promising antiviral targets against IV in *in vitro* and *in vivo* studies and clinical trials. While administration of NF-κB pathway inhibiting agents, such as LASAG results in decreased IV replication, it remained unclear whether blocking of NF-κB might sensitize cells to secondary bacterial infections, which often come along with viral infections. Thus, we examined IV and *Staphylococcus aureus* growth during LASAG treatment. Interestingly, our data reveal that the presence of LASAG during superinfection still leads to reduced IV titers. Furthermore, the inhibition of the NF-κB pathway resulted in decreased intracellular *Staphylococcus aureus* loads within epithelial cells, indicating a dependency on the pathway for bacterial uptake. Unfortunately, so far it is not entirely clear if this phenomenon might be a drawback in bacterial clearance during infection.

## Introduction

Influenza virus (IV) can cause severe respiratory illnesses. Epidemic episodes and pandemic outbreaks clearly demonstrate that this pathogen still has a strong impact on global health, which is also due to its high genetic variability and rapid formation of resistant variants against prophylactic and therapeutic agents. In addition to common treatments, such as vaccination and neuraminidase inhibition, new therapeutic strategies focusing on the inhibition of virus supportive cellular signaling pathways, are under development. Over the past decades several signaling cascades, such as the Raf/MEK/ERK, the PI3K/Akt, the PKC and the NF-κB pathways, have been identified to support IV infection [1]. With respect to viral replication, the NF-κB pathway was introduced as a promising candidate for the development of antiviral strategies. Blockage of NF-κB signaling with different inhibitors as antiviral approach has been extensively studied in the past [2–7]. Mechanistically, inhibition of virus-mediated NF-κB activation was shown to lead to impaired expression of pro-apoptotic factors such as TNF-related apoptosis inducing ligand (TRAIL) and FasL [4], resulting in subsequent inhibition of caspase activity [8]. This prevents nuclear pore degradation and therefore impairs caspase-mediated viral ribonucleoprotein export [4, 9, 10], finally resulting in reduced release of progeny virus particles. In virus-infected cells, as well as in mice, the commonly used drug acetylsalicylic acid (ASA), which has been described to specifically block the NF-κB-inducing kinase IKKβ [11] was already demonstrated to inhibit virus-mediated NF-κB activation and virus replication [3]. Furthermore, ASA treatment did not result in the development of resistant virus variants in a multi-passaging experiment [3]. The approach however, had two drawbacks: ASA is a rather unstable compound in aqueous solution and the concentrations of ASA needed for sufficient deposition in the lungs could not be reached by oral administration. The ASA salt D, L-lysine-acetylsalicylate glycine (LASAG), as a more sophisticated drug, is suitable to be applied topically as an aerosol directly into the lung. Due to its bioavailability and advantageous application form via inhalation, LASAG represents a potential antiviral drug, which was already investigated in A549 human lung epithelial cells *in vitro* as well as in mice by LASAG inhalation, resulting in efficient titer reduction and protection of mice from lethal infection [6, 12]. The LASAG approach was meanwhile also evaluated in a randomized double blinded phase II clinical trial in IV-infected patients, showing that the administration is safe and leads to a faster decline of influenza symptoms compared to standard of care [12].

IV infections in humans are often accompanied with secondary bacterial infections or even increase the susceptibility for bacterial outgrowth. The most common pathogens inducing bacterial pneumonia associated with IV are *Staphylococcus aureus* (*S*. *aureus*) and *Streptococcus pneumonia* (*S*. *pneumoniae*) [13]. A high number of fatal cases was observed during simultaneous infections with IV and *S*. *aureus*, while superinfection with *S*. *pneumoniae* typically occurred late in infection after viral clearance [14–16]. Superinfections often lead to an increase in viral as well as bacterial load and a high degree of inflammatory lung damage [16–19]. Very recently the direct interaction between IV and different bacteria were reported to promote bacterial adherence to epithelial cells [20]. Regarding further interfering mechanisms of IV infection and bacterial clearance it was shown that the viral-mediated induction of type I interferons facilitates the post-influenza bacterial infection by for example reducing the amount of chemo-attractants resulting in an impairment of neutrophil recruitment [21]. Also the increased surface presentation of α5 integrins after IV infection results in a better adherence of bacteria, pointing to the impaired bacterial clearance and the severity of superinfection [22]. This severe illness progression is the result of a complex molecular interplay of both pathogens with the host, which is not fully understood. Additionally, development of resistant IV variants and resistant bacterial strains like Methicillin-resistant *S*. *aureus* (MRSA) increase the problem to treat infections with common therapeutics. Taking the occurrence of bacterial superinfections in IV-infected individuals and the multilayered interplays of those viral and bacterial invaders into account, it is necessary to address whether the blockade of a central cell intrinsic innate immune regulator such as NF-κB as anti-IV approach may come at the cost of sensitizing lung epithelial cells to secondary bacterial infections.

In the present manuscript we investigated the cell intrinsic effect of the NF-κB inhibitor LASAG on IV and *S*. *aureus* superinfection. First of all, our experiments demonstrated a reduction of IV titers not only in single, but also in *S*. *aureus* superinfected cells after LASAG treatment. In addition, LASAG treatment did not lead to a cell intrinsic sensitization of epithelial cells for bacterial growth since bacterial loads were even decreased rather than enhanced. Finally, LASAG treatment did not provoke enhanced titers in bacterial suspension.

## Material and methods

### Ethics statement

Experiments with human blood were approved by the ethical review committee of the University Hospital Jena, Germany (license number: 2019–1519).

### Cell lines, virus strain and bacteria strains

The human lung epithelial cell line A549 [American Type Culture Collection (ATCC #CCL-185), Wesel, Germany] was cultivated and grown in Dublecco’s modified Eagle medium (DMEM; Sigma-Aldrich). The Madin-Darby canine kidney cells (MDCKII; ATCC #CRL-2936) were cultivated and grown in minimum essential medium Eagle (MEM; Sigma-Aldrich). Both media were supplemented with 10% fetal calf serum (Biochrom, Berlin, Germany). Cells were cultivated at 37 °C and 5% CO_2_.

The human IV A/Puerto Rico/8/34 (H1N1; PR8-M) [23] was propagated on 10-day-old embryonated chicken eggs.

Methicillin-sensitive *S*. *aureus* strains 6850 (ATCC #53657), SH1000 (derivative of laboratory strain 8325–4) [24] and Methicillin-resistant *S*. *aureus* strain USA300 [25] were stored at -80 °C in a 30% glycerol/brain heart infusion (BHI; Merck, Darmstadt, Germany) medium. For bacterial infection, bacteria were cultivated on blood agar plates to gain single colonies, which were taken to inoculate 5 ml BHI medium at 37 °C at 5% CO_2_ without shaking.

### Bacterial preparation

Prior to bacterial infection of A549 cells an overnight culture was prepared by inoculating 5 ml of BHI medium with a single colony taken from a blood agar plate and incubated for 16 h at 37 °C at 5% CO_2_. The bacterial culture was washed with phosphate buffered saline (PBS) and the number of bacteria was measured by determining the optical density of 1 at OD_600nm_. The corresponding bacterial concentration at an OD_600nm_ = 1 (5 × 10^8^ CFU ml^-1^) was previously determined by growth kinetics.

### Infection protocols and bacterial titer measurements

Cells were seeded in 6-well plates (A549: 0.5 × 10^6^) in 2 ml or in 12-well plates (A549: 0.1 × 10^6^) in 1 ml culture medium for 16 h.

For superinfection, cells were washed with PBS and incubated with PBS/BA (0.2% bovine serum albumin, 1 mM MgCl_2_, 0.9 mM CaCl_2_, 100 U ml^-1^ penicillin, 0.1 mg ml^-1^ streptomycin) in presence or absence of the virus (MOI = 0.1) for 30 min at 37 °C, 5% CO_2_. Afterwards, cells were rinsed with PBS and the bacterial infection was performed in DMEM/INV (1% human serum albumin, 25 nmol l^-1^ HEPES) in the presence or absence of 5 mM LASAG (Aspirin^®^, i.v. 500 mg, Bayer) at the indicated MOIs. After 3 h cells were washed with PBS and treated with lysostaphin to remove extracellular bacteria to avoid bacterial overgrowth. Therefore, cells were incubated with DMEM/FBS (10% FBS, 2 μg ml^-1^ lysostaphin) for 20 min at 37 °C. Subsequently, cells were washed with PBS and incubated with DMEM/BA (0.2% BSA, 1 mM MgCl_2_, 0.9 mM CaCl_2_, 3 ng ml^-1^ Trypsin-TPCK) in the presence or absence of 5 mM LASAG until 18 h post virus infection at 37 °C, 5% CO_2_.

To determine bacterial titers after LASAG treatment during early times of infection or of TNFα-activated cells, cells were washed with PBS and either left untreated or pre-incubated with 2.5 ng ml^-1^ TNFα in the presence or absence of 5 mM LASAG dissolved in DMEM/INV for 4 h at 37 °C at 5% CO_2_. Alternatively, 20 μM BAY-11-7085 was used for inhibition of NF-κB with DMSO as solvent control (untreated). Afterwards cells were washed with PBS and infected with bacteria at the indicated MOI in DMEM/INV for the indicated time points at 37 °C and 5% CO_2_ in the presence or absence of 2.5 ng ml^-1^ TNFα, 5 mM LASAG, 20 μM BAY-11-7085 or DMSO as solvent control (untreated). Lysostaphin treatment (2 μg ml^-1^) was performed to remove extracellular bacteria before determination of intracellular bacterial titers. Cells were washed with PBS and lysed via hypotonic shock with 2 ml of autoclaved and distilled H_2_O for 30 min at 37 °C and 5% CO_2_. Cells were scraped off and cell suspension was centrifuged at 3200 g and 4 °C for 15 min. The pellet was resuspended in 1 ml PBS, serially diluted and plated on BHI agar plates. Finally, the plates were incubated overnight at 37 °C. Each experiment was performed in three independent experiments in technical duplicates.

### Standard plaque assay

Viral titers were determined by titration of infectious viral particles in the supernatant by a standard plaque assay as described earlier [3].

### p65 siRNA knockdown and immunofluorescence microscopy

For transfection experiments, scrambled siRNA for negative control (scRNA) and p65 siRNA, both conjugated with AlexaFluor555 (purchased from ambion by life technologies; Carlsbad; USA) were introduced into A549 cells. Therefore, A549 human lung epithelial cells were freshly seeded onto coverslips in 12-well plates (0.1 ×10^6^ in 1 ml culture media). Transfection suspension was prepared by diluting 150 pmol of scRNA or p65 siRNA with 121 μl Opti-MEM (Life Technologies, Darmstadt, Germany) and 2.5 μl lipofectamine 2000 and incubated at RT for 20 min. Transfection suspension was added drop wise onto the cells and incubated for 48 h at 37 °C and 5% CO_2_. Afterwards, cells were washed with PBS and infected with *S*. *aureus* 6850-GFP (MOI = 5) for 2 h at 37 °C and 5% CO_2_, followed by PBS washing and lysostaphin treatment as described above. Subsequently, cells were fixated with 300 μl 4% paraformaldehyde (PFA) for 15 min at room temperature (RT). Cells were washed two times with PBS for 5 min at RT shaking (20 rpm). Nuclei were stained by adding 400 μl/well with DAPI (1.09 μM diluted in PBS) for 10 min at RT shaking (20 rpm). Cells were again washed two times with PBS for 5 min at RT shaking (20 rpm) before mounting coverslips with Fluorescent Mounting Medium (Dako, California, USA). Slides were stored under light exclusion for at least 20 min at RT to allow solidification of the mounting medium. For prolonged storage, the excess resin was carefully removed with ethanol, the edges of the coverslips sealed with clear coat and samples stored in a slide box at 4 °C. Daylight was avoided throughout the whole experiment. Images were acquired with ZEISS LSM 800 with Airyscan and further formatted using ZEN software (ZEISS Efficient Navigation) at 20x (Zeiss Axio Observer inverted microscope). Immunofluorescence images were analyzed with CellProfiler (v.3.0.0, Broad Institute of Harvard and MIT, England). For this purpose, the cells were first detected on the basis of the DAPI fluorescence signal of the nuclei. Non-transfected cells were excluded by detection of AlexaFluor555 signal to gain only positively transfected cells. The mean intensity of *S*. *aureus* 6850-GFP signal was determined to display values for positive-infected cells (0.25 mean intensity units).

### Quantification and statistical analysis

All data represent the means standard deviation (+ SD) of at least three independent experiments. Statistical significances were determined by one-way ANOVA followed by Tukey’s or Dunnett’s multiple comparison test or two-tailed one-sample t-test using GraphPad Prim software (v.7.03, GraphPad Prism, Inc., La Jolla, CA, USA).

## Results

The efficient blockade of IV replication with the NF-κB blocking agents ASA or its lysine-salt LASAG has been described previously [3, 6, 12]. Inhibition of cellular factors exploited by the virus has a major advantage compared to directly targeting the pathogen as the likeliness of resistance development is reduced. However, inhibition of the NF-κB pathway as a major mediator of anti-pathogen responses might sensitize host cells to secondary bacterial infections, which was in focus of interest in the present study.

### IV and *S*. *aureus* titers are reduced in single- and superinfection upon LASAG treatment

**Fig 1:**
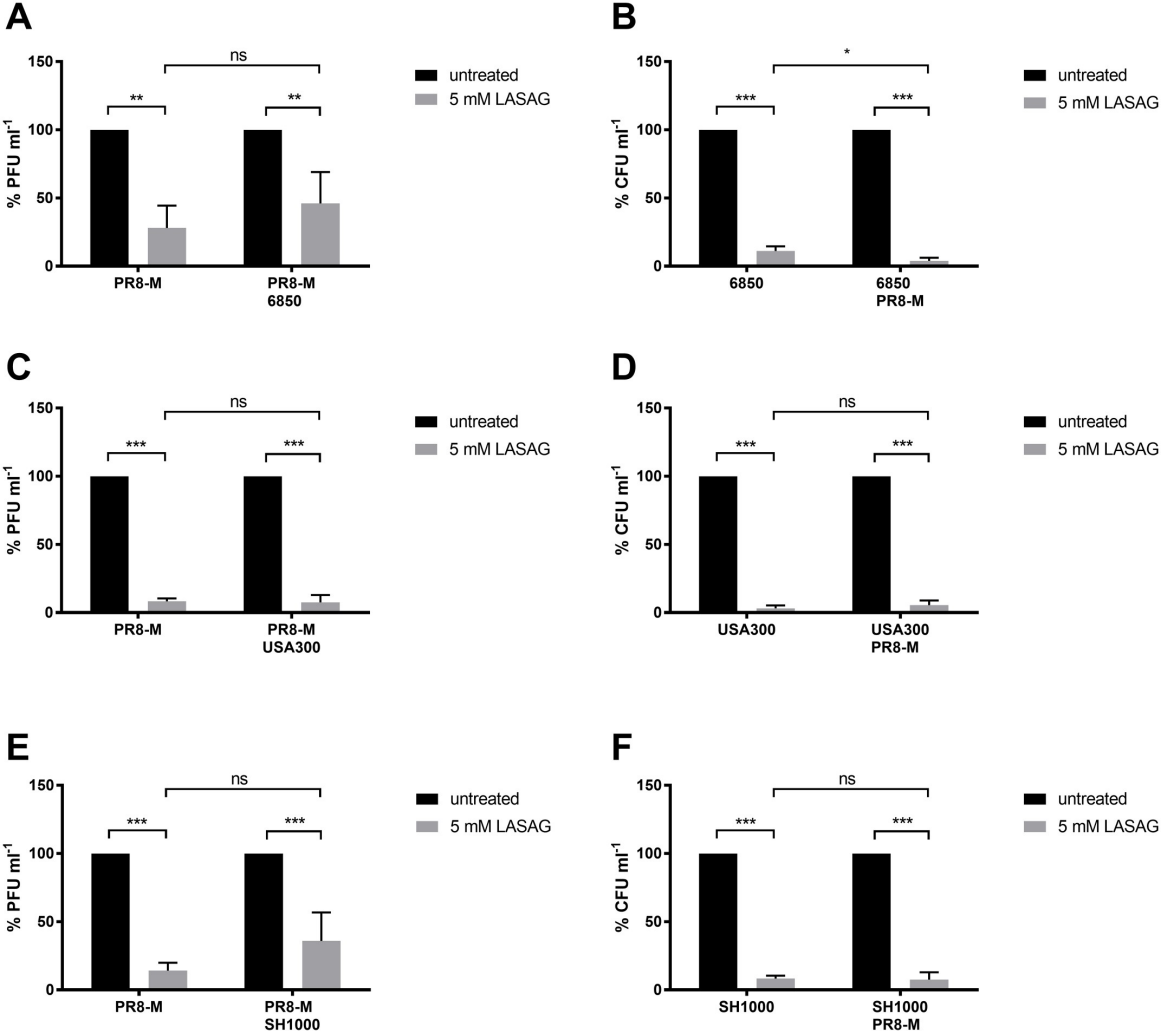
IV and *S*. *aureus* titers are reduced in single- and superinfection upon LASAG treatment. (A-F) A549 human lung epithelial cells were infected with IV PR8-M (MOI = 0.1) for 30 min at 37 °C, and/or superinfected with the *S*. *aureus* strains 6850 (MOI = 0.1), USA300 or SH1000 (MOI = 0.01) in the presence or absence of 5 mM LASAG for 3 h at 37 °C and 5% CO_2_. Lysostaphin (2 μg ml^-1^) was used to remove extracellular bacteria. Afterwards, cells were incubated in the presence or absence of 5 mM LASAG until 18 h post viral infection. Before cell lysis via hypotonic shock (30 min, 37 °C), supernatants were collected to determine viral titers. Bacterial titers were determined by plating serially diluted cell lysates. Data represent the mean + SD of three independently repeated experiments. Statistical significance was evaluated by one-way ANOVA and subsequent Tukey’s multiple comparison test (* p < 0.05; ** p < 0.01; *** p < 0.001; ns = not significant).

To elucidate the impact of LASAG treatment during a superinfection scenario we determined viral and bacterial titers upon multi-cycle IV infection *in vitro*. A549 human lung epithelial cells were infected with the IV strain A/Puerto Rico/8/34 (H1N1; PR8-M) and superinfected with the *S*. *aureus* strains 6850, USA300 and SH1000 for 18 h at the indicated multiplicity of infection (MOI) in presence and absence of LASAG. Interestingly, superinfection did not affect the efficacy of LASAG to inhibit viral replication, independent of the bacterial strain. Viral titers were decreased by 64%–90% in single- and superinfection in presence of 5 mM LASAG (Fig 1A, 1C and 1E). Intriguingly, treatment with LASAG even led to a decrease of more than 90% in intracellular bacterial titers (Fig 1B, 1D and 1F) strongly arguing against a potential sensitization of epithelial cells for enhanced bacterial propagation. The 50% effective concentration (EC_50_) of LASAG was determined to be EC_50_ = 1622 μM in single bacterial infection (USA300) or EC_50_ = 1377 μM in coinfection (USA300/PR8-M). The 50% cytotoxic concentration (CC_50_) of LASAG was CC_50_ = 76185 μM, resulting in a selectivity index (SI) of 46.97 (USA300) or 55.33 (USA300/PR8-M), respectively (S1 Fig).

The LASAG-mediated inhibition of NF-κB signaling was verified by Western Blot analysis (S2 Fig). Furthermore, a reduction of intracellular bacterial uptake into LASAG-treated primary normal human bronchial epithelial (NHBE) was detected cells (S3A Fig). Since phagocytosis is the primary bacterial clearance mechanism [26] the effect of LASAG on *S*. *aureus* uptake into human polymorphonuclear neutrophils (PMN) was further analyzed (S3B Fig). While in presence of LASAG *S*. *aureus* uptake was significantly decreased into NHBE cells (S3A Fig), correlating to the finding in immortalized A549 cells, the *S*. *aureus* internalization into PMN was not significantly reduced (S3B Fig).

In summary, these data clearly show that LASAG treatment does not cell-intrinsically sensitize immortalized or primary lung epithelial cells for bacterial infections and even more point towards the necessity of NF-κB-mediated signaling to promote intracellular bacterial load.

### Activation of NF-κB with TNFα leads to an increase in intracellular bacterial load

**Fig 2:**
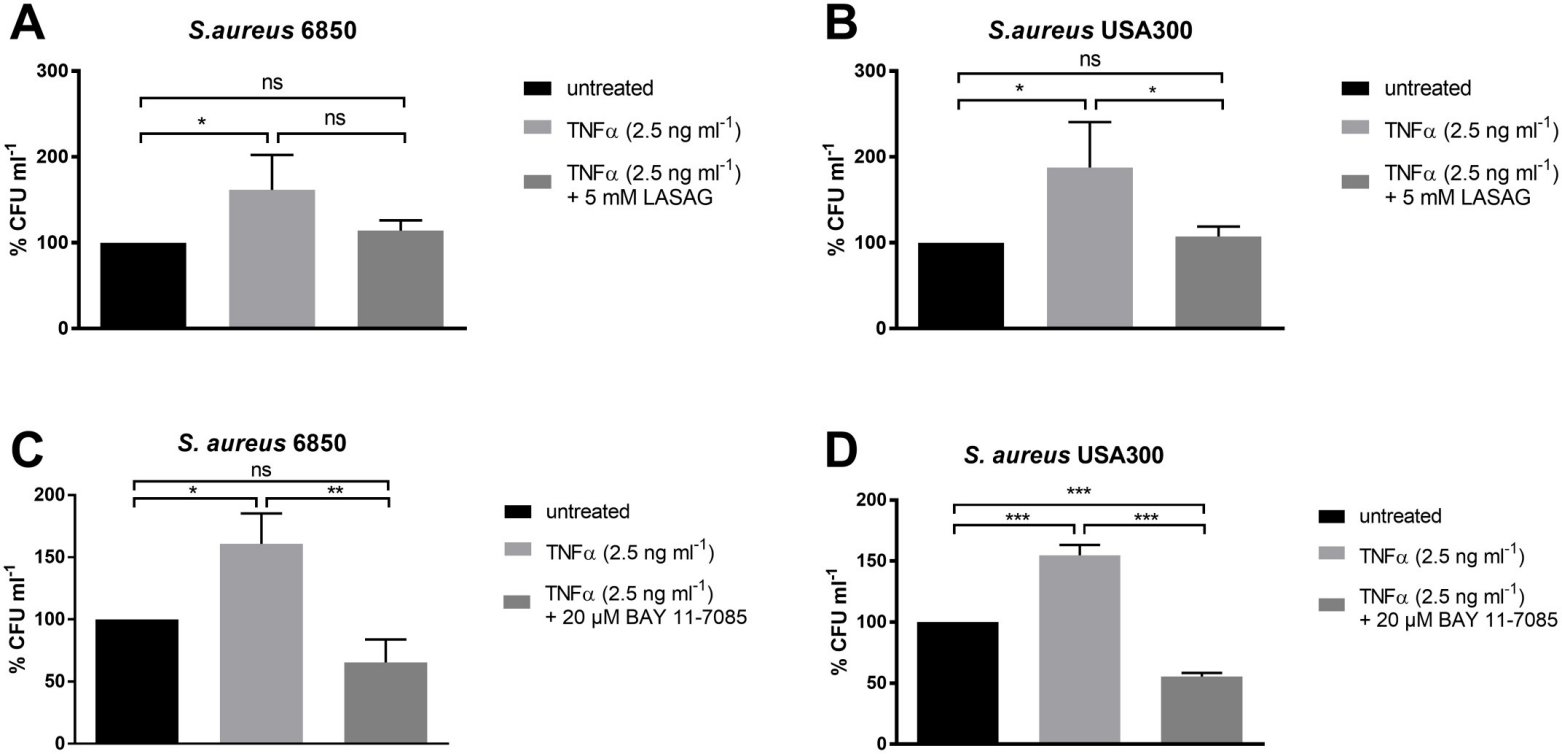
Activation of NF-κB with TNFα leads to an increase in bacterial load. (A-D) A549 human lung epithelial cells were pre-treated with 2.5 ng ml^-1^ TNFα and (A, B) 5 mM LASAG or (C, D) 20 μM BAY-701185 and DMSO as solvent control (untreated) for 4 h and afterwards infected with *S*. *aureus* 6850 or USA300 (MOI = 5) in the presence or absence of 2.5 ng ml^-1^ TNFα and (A, B) 5 mM LASAG or (C, D) 20 μM BAY-701185 and DMSO as solvent control (untreated) for 2 h at 37 °C and 5% CO_2_. Lysostaphin treatment (2 μg ml^-1^) was performed to remove extracellular bacteria. Cells were lysed via hypotonic shock to determine intracellular bacteria via plating of serial dilutions on BHI agar. Data represent the mean + SD of three independent experiments. Statistical significance was evaluated by one-way ANOVA followed by Tukey’s multiple comparison test (* p < 0.05; ** p < 0.01; *** p < 0.001; ns = not significant).

LASAG-mediated inhibition of NF-κB signaling pathway resulted in significantly reduced intracellular bacterial loads (Fig 1B, 1D and 1F), which indicates a potential function of NF-κB for intracellular uptake of *S*. *aureus*. NF-κB is a well-known regulator of cytokine and chemokine expression and can be activated by different stimuli, including tumor necrosis factor α (TNFα). Previous publications already showed an increased bacterial load in bovine endothelial cells upon TNFα-induced NF-κB activation [27]. In order to investigate if LASAG treatment affects TNFα-supported enhancement of bacterial uptake to cells, we pre-incubated A549 cells with 2.5 ng ml^-1^ TNFα for 4 h in the presence or absence of NF-κB-inhibitors and infected these cells with the Methicillin-sensitive *S*. *aureus* (MSSA) strain 6850 or Methicillin-resistant *S*. *aureus* (MRSA) strain USA300 for 120 min. Treatment with TNFα resulted in a significant increase of 61% and 87% in intracellular bacterial load of both, *S*. *aureus* 6850 and USA300 (Fig 2A and 2B). In addition to LASAG, a second structurally unrelated NF-κB inhibitor (BAY 11–7085) was used, which was previously shown to potently inhibit phosphorylation of IκBα and thus prevents activation of NF-κB [28]. Both inhibitors were able to reduce the increase in bacterial load caused by TNFα stimulation and even further decreased intracellular bacterial titers compared to untreated controls (Fig 2C and 2D).

To exclude direct effects of the inhibitors on bacterial growth we inoculated 5 ml BHI medium with increasing amounts of *S*. *aureus* 6850 in the presence and absence of 5 mM LASAG, 10 mM LASAG, 10 μM BAY 10–7085 or 20 μM BAY 10–7085 (S4 Fig). Our results clearly show that none of the used concentrations of the inhibitors have an impact on bacterial growth in a host cell free environment.

These results indicate that NF-κB activation has a beneficial effect on intracellular *S*. *aureus* load in human lung epithelial cells that is abolished after treatment with NF-κB inhibitor.

### Knockdown of p65 in A549 cells leads to decreased infection rate

**Fig 3:**
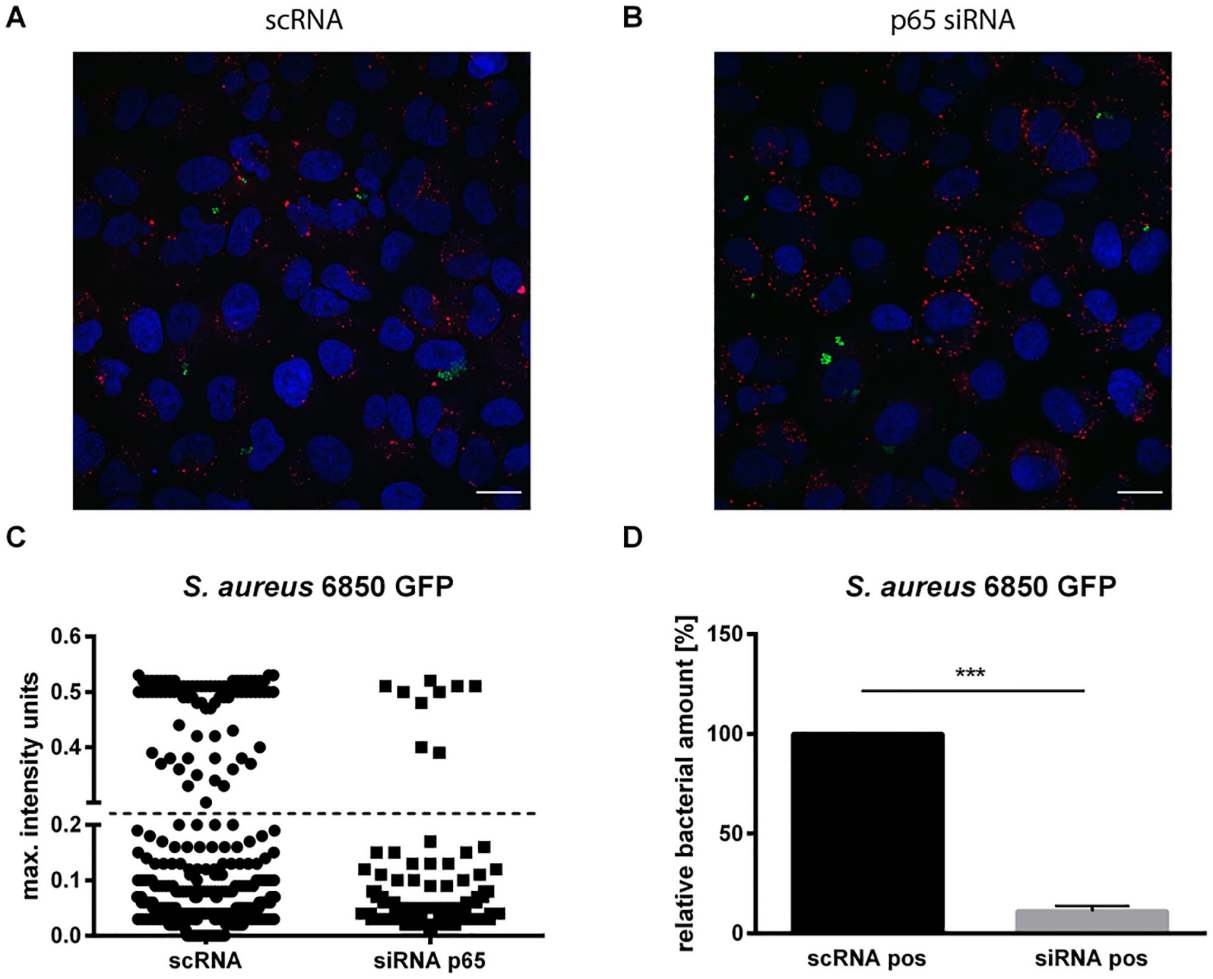
Knockdown of p65 in A549 cells results in a decreased infection rate. A549 human lung epithelial cells were transfected with scrambled control siRNA-AlexaFluor555 (scRNA) or p65-siRNA-AlexaFluor555 for 48 h on cover slips before infection with *S*. *aureus* 6850-GFP (MOI = 5). 2 h post bacterial infection, cells were treated with lysostaphin (2 μg ml^-1^) to remove extracellular bacteria and fixated with 4% PFA. (A-B) Representative images of scRNA and p65 siRNA-transfected cells (48 h) infected with *S*. *aureus* 6850-GFP (2 h), which were subjected to confocal microscope in 63x (oil immersion) magnification, are depicted (nuclei: blue/DAPI, bacteria: green/GFP, scRNA or siRNA: red/AlexaFluor555) (Scale bar, 20 μm). To identify the fluorescence signal of the nuclei for cell counts with CellProfiler, images with 20x magnification were used. 2630 positively transfected cells (1643 scRNA and 987 siRNA) were detected by identification of the AlexaFluor555 fluorescence signal. These positively transfected cells were further investigated for bacterial infection, by measuring the mean intensity units (average pixel intensity of *S*. *aureus* 6850-GFP in positively transfected cells) of GFP signals. (C) Maximum intensity units of positively transfected cells. A maximum intensity of 0.25 units indicates bacteria-infected cells. (D) Relative bacterial amount of infectivity in transfected cells. Data represent the means + SD of three independent experiments. Statistical significance was evaluated by a two-tailed one-sample t-test (*** p < 0.001).

We showed that NF-κB inhibition does not sensitize lung epithelial cells for a secondary bacterial infection after IV infection (Fig 1B, 1D and 1F) but NF-κB activation rather seems to be pro-bacterial (Fig 2). To confirm a specific NF-κB-mediated effect on bacterial infection we used p65 siRNA in a knockdown approach. We determined uptake of GFP-tagged *S*. *aureus* 6850 (6850-GFP) in p65 siRNA-transfected A549 cells in comparison to control siRNA (scRNA)-transfected cells. The infection rate of positively transfected cells was quantified by confocal laser scanning microscopy and subsequently analyzed with the cell image analysis software CellProfiler (http://cellprofiler.org) (Fig 3A–3D). Efficient knockdown of p65 was validated by Western Blot analysis (S5 Fig). To exclude presence of extracellular attached and non-internalized bacteria an antibiotic wash step was preformed to lyse extracellular bacteria prior to fixation. Exemplarily, two images are shown (Fig 3A and 3B). Our data demonstrate a clear reduction of intracellular bacterial load in p65 siRNA-transfected cells (Fig 3C). Additionally, we also compared the relative bacterial amount in control cells to p65 knockdown cells and observed a reduction of about 90% of intracellular bacteria (Fig 3D).

In summary, we were able to exclude that NF-κB inhibition by LASAG would sensitize host cells for secondary infections with *S*. *aureus*, both in co-infected immortalized and primary human lung epithelial cells. We rather observed a reduction of bacterial titers in super- and single-infection scenarios.

## Discussion

Viruses by definition are strictly dependent on host cells for their replication. This indissoluble dependency separates viral infections from e.g. bacterial or fungal pathogens. Historically, treatments against viruses have been developed to directly attack crucial viral proteins [29]. Undoubtedly, such approaches have had great success and helped to safe millions of lives around the globe [30]. Nevertheless, this strategy often came with the cost of the emergence of resistant virus variants rendering the treatment to less effective or even completely useless. A more recent strategy to fight viral invaders is to no longer attack viral structures but block necessary cellular processes during the viral life cycle [31,32]. Viruses have to cross several cellular barriers during their life cycle and especially viruses replicating in the nucleus of a host cell have to do so multiple times. These transport processes through the plasma membrane and the nuclear envelope are crucial for virus replication and are carried out by host cell factors, which are hijacked by the virus for its own purposes. One such cellular factor, which has been shown to be necessary for IV propagation is the NF-κB signaling module [4]. The antiviral capacity of NF-κB inhibition against IV infections has been studied in great detail within the last two decades [2–4, 12]. However, the IV infection scenarios seen by physicians in the clinics are often diverse and for IV infections in particular, bacterial co-infections can become a very critical complication [15]. NF-κB furthermore is described as a crucial cell intrinsic regulator of inflammatory processes by driving the expression of multiple inflammatory genes and thereby activating several anti-pathogen networks [33], which may make the use of NF-κB inhibitors such as LASAG counterintuitive. Even more, it has already been shown that the coincidence of viral and bacterial infections alters cell death mechanisms, cytokine expression and pathogen titers [34,35]. The functions of ASA in immune responses were discussed controversial. ASA was described as a modulator of phagocytosis of PMN and dendritic cells [36,37] but also as inhibitor of LPS-induced macrophage activation improving inflammatory conditions [38]. Therefore, it is of importance to understand potential consequences of this antiviral treatment for bacterial co-invaders to avoid any kind of cell intrinsic sensitization of lung epithelial cells for secondary bacterial infection by blocking NF-κB. Thus, we investigated if LASAG treatment does sensitize lung epithelial cells to bacterial superinfection after an initial IV infection. The data showed no increase in bacterial load arguing against any cell-intrinsic sensitization for superinfections after LASAG treatment. In addition, the antiviral effect of LASAG was still detectible in *S*. *aureus* superinfected samples confirming that LASAG treatment does block IV replication also in the presence of a co-invading bacterial pathogen. To our surprise, instead of an increase, bacterial loads were rather decreased in LASAG-treated samples (Fig 1). These results give rise to the assumption that NF-κB activity is actually required for efficient *S*. *aureus* infections of human lung epithelial cells as well. It was already described that bacterial pathogens manipulate kinase-signaling cascades of the host cell for their propagation. While inhibition of NF-κB was shown to down regulate innate immune responses in host cells, the activation of this signaling pathway resulted in the transcription of surface proteins for better attachment and internalization [39]. In case of *S*. *aureus* the activity state of NF-κB was also described to have an impact on intracellular bacterial load [27]. The effect of NF-κB activation after *S*. *aureus* infection was previously reported [40] but there is an urgent need for additional investigations. To get further insights into this potential interplay of *S*. *aureus* infectivity and host cell NF-κB signaling, we validated the impact of NF-κB on intracellular bacterial titers. Respectively, TNFα, a strong activator of NF-κB signaling, promoted *S*. *aureus* load. Although TNFα-induced NF-κB activity was correlated with an increased intracellular bacterial load, we were able to reverse this effect by use of the NF-κB inhibitors LASAG and BAY 11–7085 (Fig 2). To further confirm a specific function of NF-κB in bacterial infection, we performed knockdown experiments of p65 to analyze *S*. *aureus* titers in siRNA-transfected A549 human lung epithelial cells via immunofluorescence. After knockdown of p65, a clear reduction of intracellular bacteria was detected (Fig 3), verifying the exploitation of the NF-κB signaling pathway by *S*. *aureus* to a further extent.

Taken together, our experiments exclude a sensitization of IV-infected A549 human lung epithelial cells and primary normal human bronchial epithelial cells for *S*. *aureus* internalization by using LASAG. Concomitantly, our data indicate a non-significant but slightly reduced uptake into polymorphonuclear neutrophils, which might be a drawback during bacterial clearance. Thus, due to the use of mono-cell culture systems within the present study, it cannot be entirely excluded that treatment of IV-infected individuals with LASAG is contraindicated. In consequence more complex *in vivo* analysis are required to transfer our findings to a living organism. Nonetheless, since it is known that IV infection results in enhanced tissue damage, which paves the way for secondary bacterial infection and deregulation of immune response [15,17–19], the primary reduction of IV load is supposed to be beneficial for the host. Furthermore, direct interactions between IV and bacteria, promoting bacterial adherence to respiratory cells would be lessened. Due to the depicted effects of LASAG against bacterial internalization into epithelial cells, this treatment approach can be seen as fundament for potential advanced developments of LASAG in therapy of IV and secondary bacterial infections. Provided that *S*. *aureus* cannot internalize into epithelial cells to hide from the immune system in presence of LASAG, a combinatory antibiotic therapy might be an efficient alternative. Beyond that, LASAG is also reported to prevent infection-induced coagulopathy and associated tissue damage [41]. Thus, its administration during secondary bacterial infection might be another benefit. Beyond doubt, further investigations are urgently needed to verify LASAG as a promising candidate as an anti-pathogenic agent in superinfections of IV and *S*. *aureus*.

## Supporting information

S1 Fig The 50% effective concentration of LASAG in single bacteria or superinfection. (A-D) A549 human lung epithelial cells were left uninfected or were infected with IV PR8-M (MOI = 0.1) for 30 min at 37 °C, and/or superinfected with the *S*. *aureus* USA300 (MOI = 0.01) in the presence and absence of the increasing concentrations of LASAG for 3 h at 37 °C and 5% CO_2_ (A and B). To remove extracellular bacteria lysostaphin treatment was included (2 μg ml^-1^). Afterwards, cells were incubated in the presence or absence of LASAG at the indicated concentrations until 18 h post viral infection. Before cell lysis via hypotonic shock (30 min, 37 °C), cells were washed with PBS. Bacterial titers were determined upon serial dilution of cell lysates on agar plates. Bacterial titers (A, B) as well as the calculation of 50% effective concentration of LASAG (C, D) of either single *S*. *aureus* USA300 infection (A, C) or *S*. *aureus* USA300 and PR8-M superinfection (B, D) are shown. (E) A549 cells were treated with the same concentrations of LASAG indicated in (A-D) and incubated for 18 h. Supernatants were collected to analyze the LDH release and determine the 50% cytotoxic concentration (CC_50_). Data represent the mean +SD (A, B) and ± SD (C–E) of three independent experiments. Statistical significance was evaluated by one-way ANOVA followed by Dunnett’s multiple comparison test (A, B) (* p < 0.05; ** p < 0.01).
(TIF)

S2 Fig LASAG-mediated inhibition is NF-κB dependent. A549 human lung epithelial cells were left uninfected or were infected with IV PR8-M (MOI = 0.1) and/or superinfected with the *S*. *aureus* 6850 (MOI = 0.1) as described in the material and method section. After infection, cells were lysed to perform Western Blot analysis. Monitored are the protein amounts of phospho-p65, IV M1 and *S*. *aureus* PGN. ERK-2 served as loading control.
(TIF)

S3 Fig Bacterial uptake is significantly reduced in primary bronchial epithelial cells upon LASAG treatment in comparison to neutrophils. (A) Human primary epithelial cells (NHBE) were cultivated for five days and infected with *S*. *aureus* USA300-GFP (MOI = 5) for 90 min in the presence or absence of 5 mM LASAG. To remove extracellular bacteria lysostaphin treatment was included (2 μg ml^-1^). Cells were further incubated in presence or absence of 5 mM LASAG for 3 h. Afterwards, cells were detached with Accutase solution, fixated and resuspended in staining buffer for FACS analysis. (B) Human polymorphonuclear neutrophils (PMN) were isolated according to the protocol of PolymorphPrep^™^ (Progen). Cells were infected with *S*. *aureus* USA300-GFP (MOI = 5) for 90 min in the presence or absence of 5 mM LASAG. Cells were centrifuged (250 g; 8 min) and further incubated for 20 min at 37 °C and 5% CO2 in RPMI-1640 (supplemented with 10% FCS) in the presence or absence of 5 mM LASAG. (A-B) Mean fluorescence intensities (MFI) of four independent experiments are shown. Statistical significance was evaluated by using one-way ANOVA and Tukey´s multiple comparisons test (**** p < 0.0001, ns = not significant).
(TIF)

S4 Fig The NF-κB inhibitors have no impact on *S*. *aureus* 6850 growth in a cell free system. 5 ml of BHI medium were inoculated with the indicated CFU ml^-1^ in the presence or absence of 5 mM LASAG, 10 mM LASAG, 10 μM BAY 10–7085 or 20 μM BAY 10–7085 and incubated at 37 °C and 5% CO_2_ for 16 h. Bacterial cultures were centrifuged (4000 rpm; 4 °C; 10 min) and the pellets were resuspended in 1 ml PBS each. To determine bacterial titers, suspensions were serial diluted and plated on BHI agar. Data represent the mean + SD of three independent experiments. Statistical significance was evaluated by one-way ANOVA followed by Tukey’s multiple comparisons test (ns = not significant).
(TIF)

S5 Fig Efficient knockdown of p65 via siRNA transfection. A549 human lung epithelial cells were transfected with scrambled control siRNA-AlexaFluor555 (scRNA) or p65-siRNA-AlexaFluor555 for 48 h in a 12-well plate before infection with *S*. *aureus* 6850-GFP (MOI = 5). 2 h post bacterial infection, cells were treated with lysostaphin (2 μg ml^-1^) to remove extracellular bacteria. After infection, cells were lysed to perform Western Blot analysis. Monitored are the protein amounts of p65 and ERK 1/2 as loading control.
(TIF)
