# Supplementary figures and images for: The influenza replication blocking inhibitor LASAG does not sensitize human epithelial cells for bacterial infections
Source: PLoS One. 2020 May 15;15(5):e0233052. doi: 10.1371/journal.pone.0233052 (PMC7228112; doi:10.1371/journal.pone.0233052)

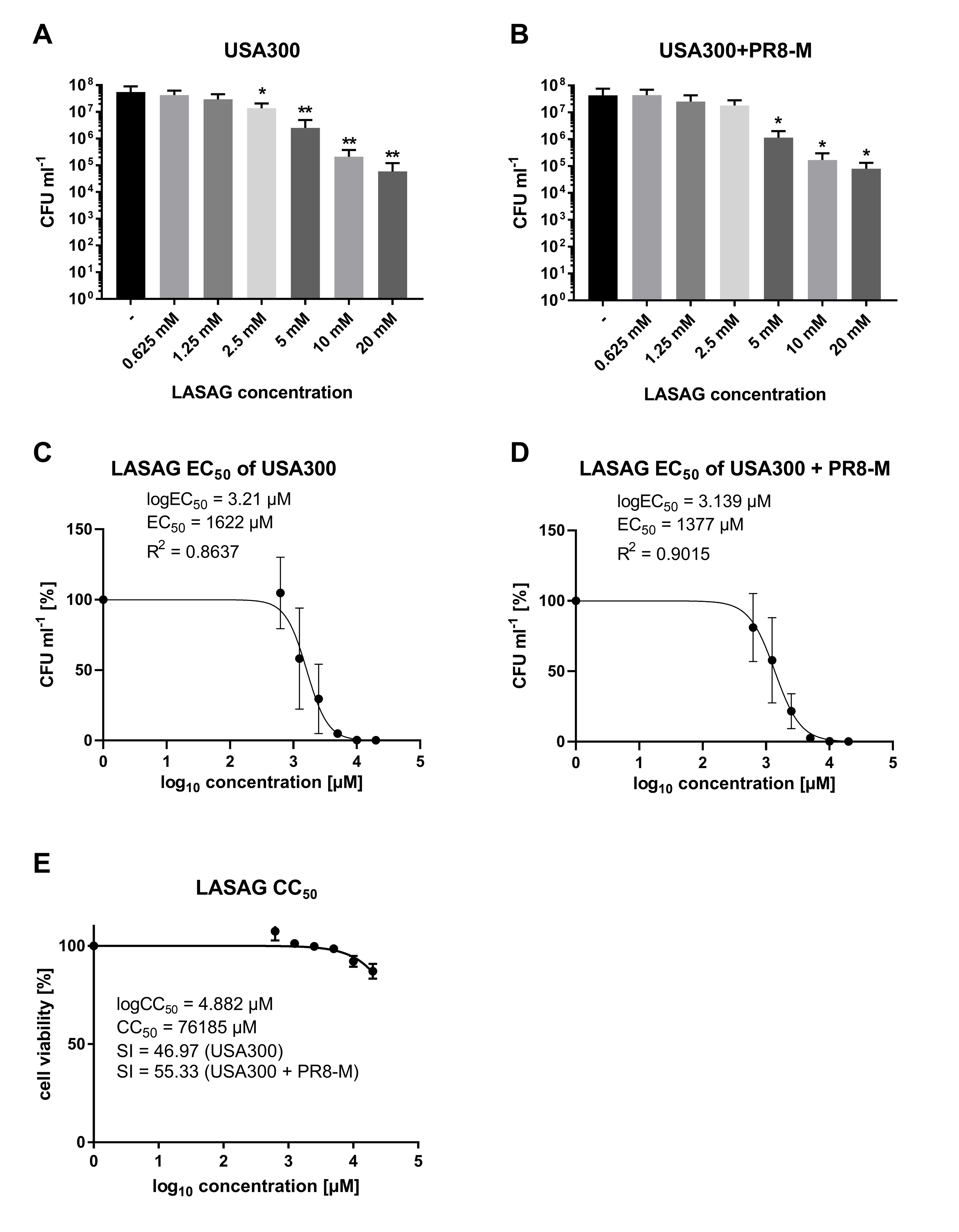

Supplement: S1 Fig — (A-D) A549 human lung epithelial cells were left uninfected or were infected with IV PR8-M (MOI = 0.1) for 30 min at 37 °C, and/or superinfected with the S. aureus USA300 (MOI = 0.01) in the presence and absence of the increasing concentrations of LASAG for 3 h at 37 °C and 5% CO2 (A and B). To remove extracellular bacteria lysostaphin treatment was included (2 μg ml-1). Afterwards, cells were incubated in the presence or absence of LASAG at the indicated concentrations until 18 h post viral infection. Before cell lysis via hypotonic shock (30 min, 37 °C), cells were washed with PBS. Bacterial titers were determined upon serial dilution of cell lysates on agar plates. Bacterial titers (A, B) as well as the calculation of 50% effective concentration of LASAG (C, D) of either single S. aureus USA300 infection (A, C) or S. aureus USA300 and PR8-M superinfection (B, D) are shown. (E) A549 cells were treated with the same concentrations of LASAG indicated in (A-D) and incubated for 18 h. Supernatants were collected to analyze the LDH release and determine the 50% cytotoxic concentration (CC50). Data represent the mean +SD (A, B) and ± SD (C–E) of three independent experiments. Statistical significance was evaluated by one-way ANOVA followed by Dunnett’s multiple comparison test (A, B) (* p < 0.05; ** p < 0.01). (TIF) [file pone.0233052.s001.tif]

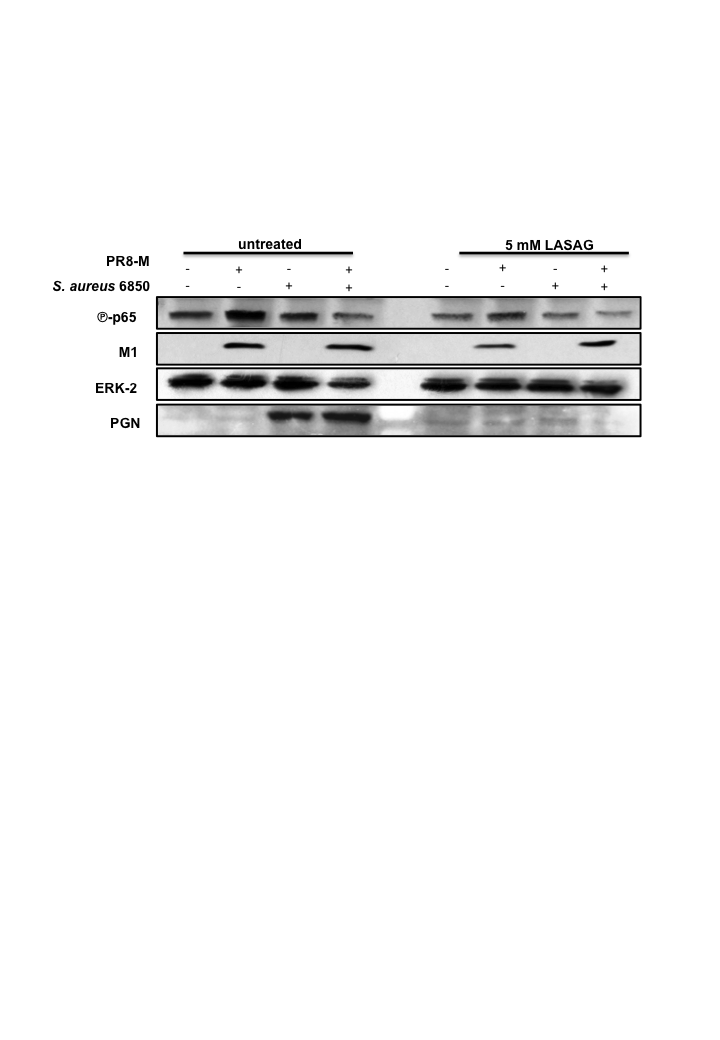

Supplement: S2 Fig — A549 human lung epithelial cells were left uninfected or were infected with IV PR8-M (MOI = 0.1) and/or superinfected with the S. aureus 6850 (MOI = 0.1) as described in the material and method section. After infection, cells were lysed to perform Western Blot analysis. Monitored are the protein amounts of phospho-p65, IV M1 and S. aureus PGN. ERK-2 served as loading control. (TIF) [file pone.0233052.s002.tif]

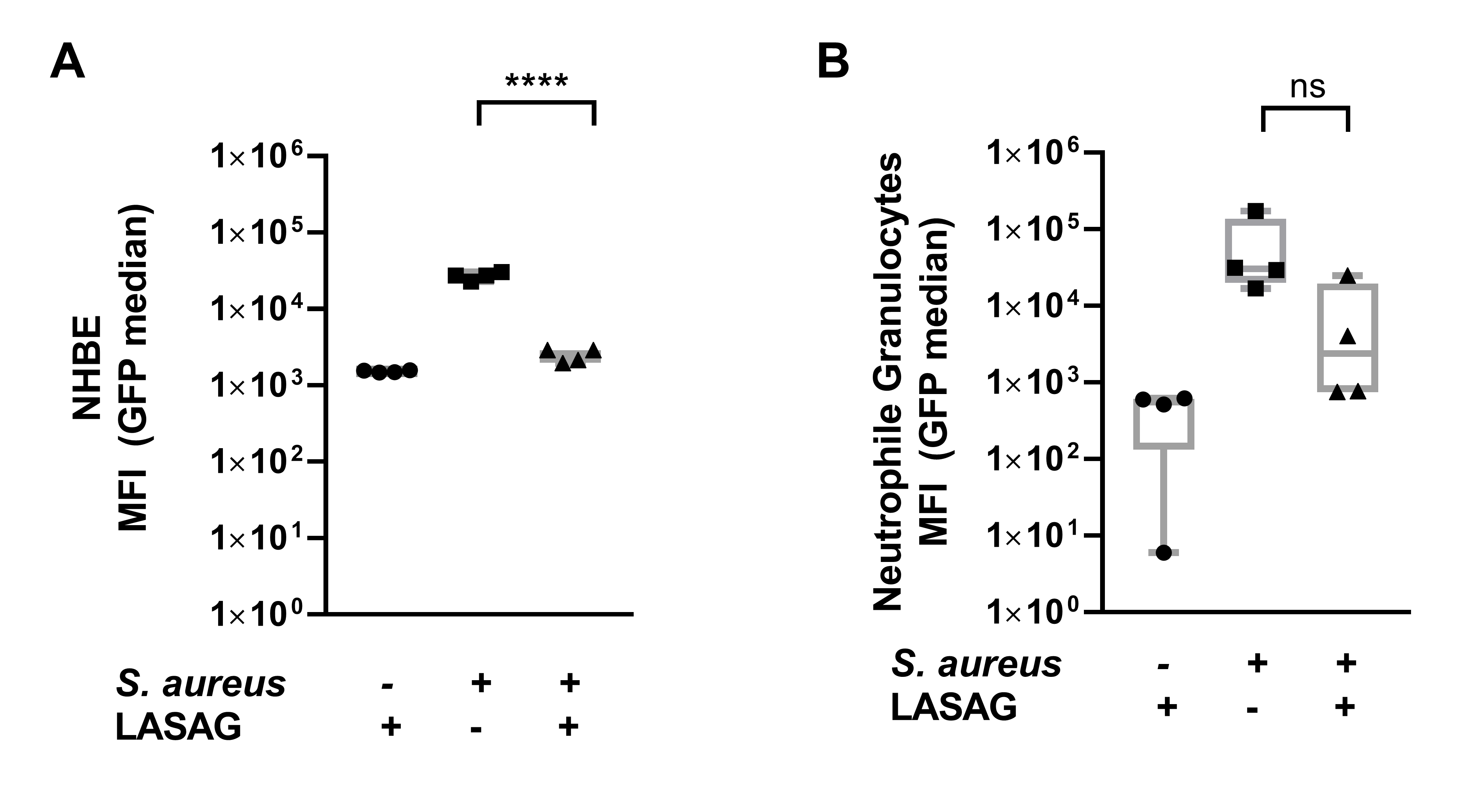

Supplement: S3 Fig — (A) Human primary epithelial cells (NHBE) were cultivated for five days and infected with S. aureus USA300-GFP (MOI = 5) for 90 min in the presence or absence of 5 mM LASAG. To remove extracellular bacteria lysostaphin treatment was included (2 μg ml-1). Cells were further incubated in presence or absence of 5 mM LASAG for 3 h. Afterwards, cells were detached with Accutase solution, fixated and resuspended in staining buffer for FACS analysis. (B) Human polymorphonuclear neutrophils (PMN) were isolated according to the protocol of PolymorphPrep™ (Progen). Cells were infected with S. aureus USA300-GFP (MOI = 5) for 90 min in the presence or absence of 5 mM LASAG. Cells were centrifuged (250 g; 8 min) and further incubated for 20 min at 37 °C and 5% CO2 in RPMI-1640 (supplemented with 10% FCS) in the presence or absence of 5 mM LASAG. (A-B) Mean fluorescence intensities (MFI) of four independent experiments are shown. Statistical significance was evaluated by using one-way ANOVA and Tukey´s multiple comparisons test (**** p < 0.0001, ns = not significant). (TIF) [file pone.0233052.s003.tif]

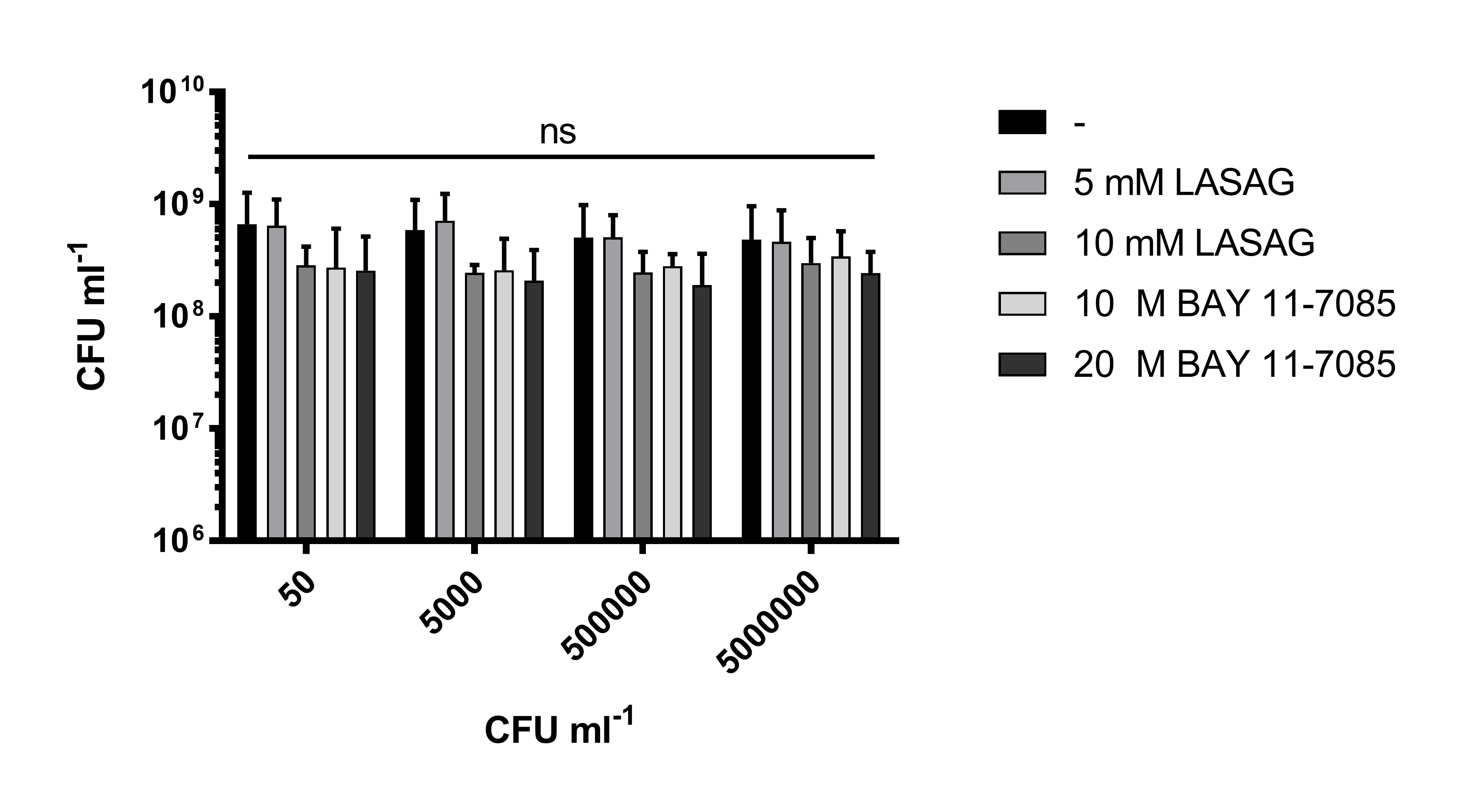

Supplement: S4 Fig — 5 ml of BHI medium were inoculated with the indicated CFU ml-1 in the presence or absence of 5 mM LASAG, 10 mM LASAG, 10 μM BAY 10–7085 or 20 μM BAY 10–7085 and incubated at 37 °C and 5% CO2 for 16 h. Bacterial cultures were centrifuged (4000 rpm; 4 °C; 10 min) and the pellets were resuspended in 1 ml PBS each. To determine bacterial titers, suspensions were serial diluted and plated on BHI agar. Data represent the mean + SD of three independent experiments. Statistical significance was evaluated by one-way ANOVA followed by Tukey’s multiple comparisons test (ns = not significant). (TIF) [file pone.0233052.s004.tif]

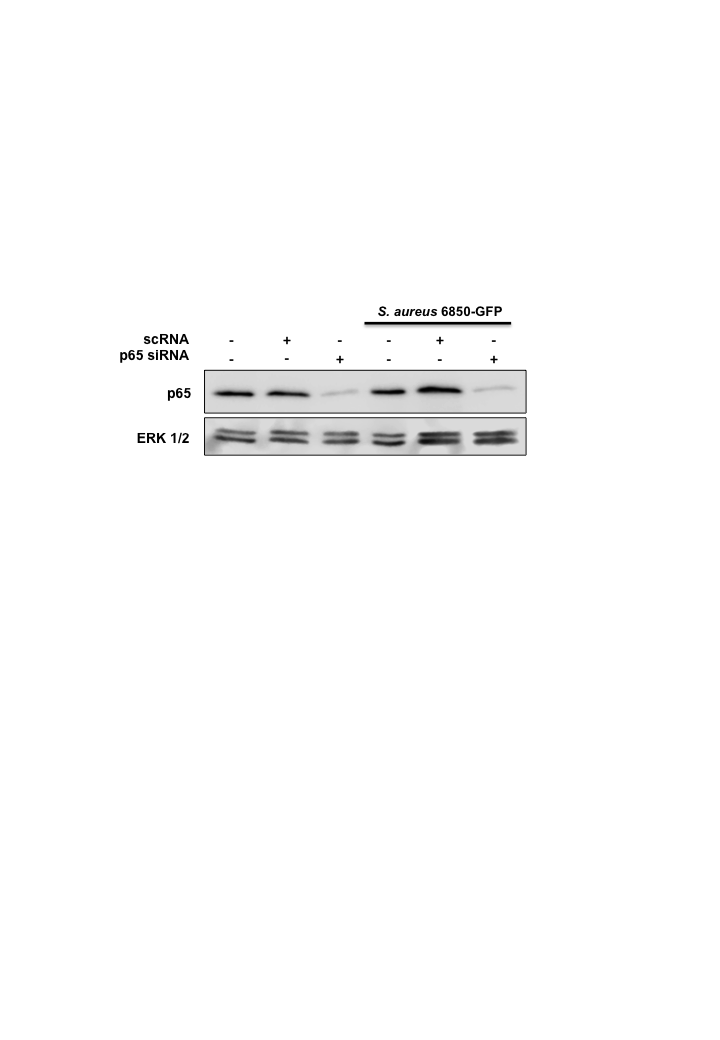

Supplement: S5 Fig — A549 human lung epithelial cells were transfected with scrambled control siRNA-AlexaFluor555 (scRNA) or p65-siRNA-AlexaFluor555 for 48 h in a 12-well plate before infection with S. aureus 6850-GFP (MOI = 5). 2 h post bacterial infection, cells were treated with lysostaphin (2 μg ml-1) to remove extracellular bacteria. After infection, cells were lysed to perform Western Blot analysis. Monitored are the protein amounts of p65 and ERK 1/2 as loading control. (TIF) [file pone.0233052.s005.tif]
